# Toward COVID-19 Contact Tracing though Wi-Fi Probes

**DOI:** 10.3390/s22062255

**Published:** 2022-03-14

**Authors:** Xu Yang, Chenqi Shi, Peihao Li, Yuqing Yin, Qiang Niu

**Affiliations:** School of Computer Science and Technology, China University of Mining and Technology, Xuzhou 221000, China; yang_xu@cumt.edu.cn (X.Y.); lipeihao@cumt.eud.cn (P.L.); yinyuqing@cumt.edu.cn (Y.Y.); niuq@cumt.edu.cn (Q.N.)

**Keywords:** COVID-19, close contact, WiFi probe requests

## Abstract

COVID-19 is currently the biggest threat that challenges all of humankind’s health and property. One promising and effective way to control the rapid spreading of this infection is searching for primary close contacts of the confirmed cases. In response, we propose COVID-19 Tracer, a low-cost passive searching system to find COVID-19 patients’ close contacts. The main idea is utilizing ubiquitous WiFi probe requests to describe the location similarity, which is then achieved by two designed range-free judgment indicators: location similarity coefficient and close contact distance. We have carried out extensive experiments in a school office building, and the experimental results show an average accuracy of more than 98%, demonstrating our system’s effectiveness in judging close contacts. Last but not least, we have developed a prototype system for a school building to find potential close contacts.

## 1. Introduction

COVID-19 is currently the biggest threat that challenges all of humankind’s health and property [1]. As of 12 May 2021, there are 159.3 million cases worldwide, including 3.3 million deaths cumulatively. Governors and researchers have been making efforts to seek exit strategies from lockdown, since although lockdown helps reduce the spread with breathtaking speed, it comes at the expense of society’s deployment. From the successful lesson of China containing the epidemic, it is believed that tracing and isolating the close contacts of infected people is no doubt a promising and effective way to prevent onward transmission [2,3]. In this paper, we aim to explore researching close contacts of a confirmed case via smartphones and WiFi probes.

A close contact is usually considered a person who had face-to-face contact or shared adjacent areas with the confirmed case outdoors or indoors [4,5]. We can obtain close contact information through tracking one’s smartphone and obtaining the movement routine through mobile communication base stations or GPS modules, but the situation is unsatisfactory in an indoor environment because of the occlusion and severe multi-path effect. Thus, searching for close contacts in the indoor environment is more critical and challenging than that in an outdoor environment. Moreover, personal privacy is another inevitable issue. Recently, Apple Inc. and Google Inc. jointly launched an application that works by broadcasting personal information through Bluetooth technology for close contact tracing [6,7,8]. Smartphones carried by users may log the information about encountering people within a certain range. Once a case gets close to the user, the smartphone can judge the status of the close-by contact based on historical data and make alarms [9,10,11]. However, this application is not well popularized due to not only the need of installing a specialized app but also the risk of personal privacy leakage.

In response to the above issues, this paper proposes a novel system named COVID-19 Tracer, aiming at using ubiquitous WiFi probe requests in the indoor environment to search the COVID-19 close contacts passively. As revealed in a survey [12,13], 75% of people may use public WiFi access. Besides, WiFi probe requests are still detectable by the access point (AP) without users connecting to WiFi routers [4]. Consequently, we can tell that our method utilizing WiFi probe requests can cover most smartphone users and provide a convenient user experience. Motivated by smartphones broadcasting probe requests that include received signal strength indicators (RSSI) and MAC addresses to seek nearby APs at all times, our method is designed to extract and calculate the location from probe information [14]. The basic idea of the probe method is that during a certain time period, if several MAC addresses would be collected by the same AP, it can be concluded that the corresponding users had been in a close area at the same time. Due to the property of probe requests, the probe approach can be realized without high-cost deployment (i.e., cameras) and user intervention (i.e., carrying wearable sensors or installing particular mobile applications) [15,16,17]. Nevertheless, this coarse-grained information results in that only the judgment of close users would benefit a lot, and there is still a further step to obtain fine-grained information about the position of a close contact to the COVID-19 case.

Considering that an AP can be accessed dozens of meters away or through floors, close contacts judgment only based on MAC addresses may lead many fault detection, i.e., people not encountering the case face-to-face, not being in the same room, and not even staying on the same floor, which may cause many unnecessary quarantines and expenses. It is necessary to specify two problems about the fine-grained information of close contacts, namely: (1) has he/she been in the same room with a confirmed case? and (2) has he/she had a face-to-face meeting with a confirmed case? Although WiFi probes provide coarse-grained RSSI, they do not profit from the existing RSSI-based indoor positioning algorithms because of the severe multi-path effects and fluctuating values. ***Therefore, it is a big challenge for COVID-19 Tracer to extract the fine-grained information of close contacts from the coarse-grained RSSI.***

In response to this issue, we propose a range-free judgment algorithm to describe location similarity with two designed judgment indicators. The first one, location similarity coefficient, utilizes the distance relationship between smartphones and surrounding APs to define the location similarity between one target patient and one other passerby. Specifically, for each smartphone, we can obtain one distance sequence of multiple APs from near to far according to the monotonically attenuating RSSI values. Then, inspired by the work [18], the relative distance between two smartphones is achieved. The second indicator is close contact distance, which calculates the absolute distance between any two smartphones with respect to the nearest AP [19]. Finally, we design a novel fine-grained judgment method for determining close contacts. We summarize the main contributions of this paper as follows:

- As far as we know, this is the first time a searching solution for COVID-19 close contacts has been provided by utilizing ubiquitous WiFi probe requests. We believe that our design can help contain the onward spreading of the virus in a low-cost effective way.
- We present a novel range-free algorithm to judge whether two people are close contacts by deriving the distance relationship between smartphones and APs.
- A series of experiments have been conducted in a school office building, and the results reveal that our method has good performance in judging close contacts with an average accuracy of more than 98%.

## 2. Preliminary

### 2.1. Wireless Probe Request

**Figure 1:**
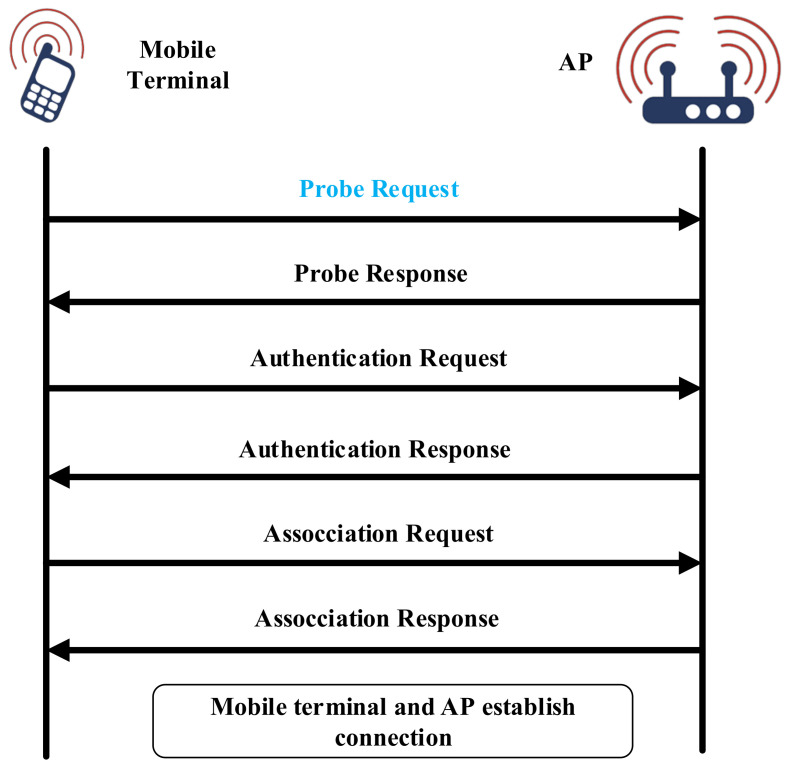
WiFi device connection process.

The wireless probe request is a necessary step to establish the WiFi connection. The WiFi connection process is shown in Figure 1. As the entrance of the network connection, the AP will continuously broadcast beacon frames to facilitate the terminal to obtain beacon frames. The terminal receives the broadcast beacon frame and obtains relevant information about the AP. Then the terminal will send probe requests to all detected APs to test whether it can establish a stable connection with the AP. After receiving the request, the AP will send a probe response to the terminal, which means that the channel between the terminal and the AP is stable and available. When the terminal needs to verify an AP, it will send an identity authentication request to the AP, and then the AP sends an authentication response to the terminal indicating that it accepts identity authentication. After the above preparations are completed, the terminal sends a connection request to the AP to request WiFi access, and then the AP replies with a connection response. Finally, the terminal establishes a connection with the AP, and the terminal successfully connects to the network and can transmit data. The wireless probe request [20] can be used by the smartphone to discover the available APs around it. In this paper, the COVID-19 Tracer determines whether two people are close contacts by mainly leveraging the MAC address and RSSI information extracted from the probe request frame.

### 2.2. Definition of Close Contacts of COVID-19

**Figure 2:**
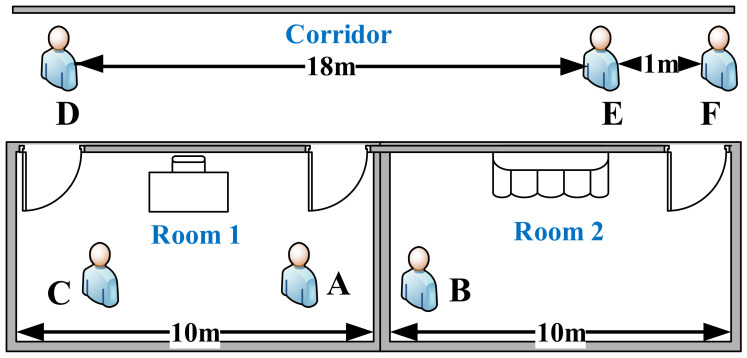
Definition of close contacts.

To begin, we have to clarify the standard definition of close contacts in this paper. During a short time period, if two people are within 5 m of each other in a corridor or in the same room, they are defined as close contacts. For instance, in Figure 2, E,F and A,C are close contacts, but *A* and *B* are not close contacts as even though they are very close, they are separated by a wall. In addition, since *D* and *E* are far away from each other, they are not close contacts.

## 3. Design

### 3.1. System Overview

**Figure 3:**
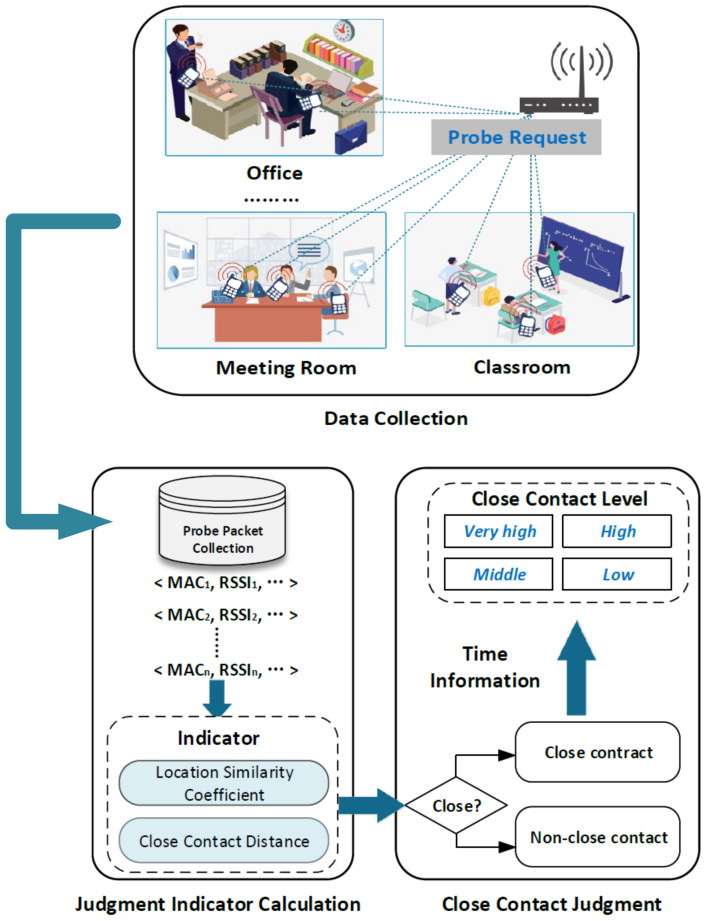
System framework.

In this section, we outline the framework of the COVID-19 Tracer. As shown in Figure 3, the COVID-19 Tracer includes three parts: data collection, judgment indicator calculation and close contact judgment.

- **Data Collection:** The smartphones continuously send out probe request frames to scan the existing 802.11 network in order to link to the wireless router devices they have previously connected. There are currently a large number of wireless routers in offices, meeting rooms, classrooms and other indoor scenes. Therefore, we can use these routers to collect probe request frames from surrounding smartphones.
- **Judgment Indicator Calculation:** With the collected probe request frames, we can obtain the relevant information from smartphones including MAC address and RSSI. Based on the RSSI information of the same smartphone from different APs, we can obtain the distance sequence reflecting the near–far relationship from one smartphone to different APs. Utilizing this sequence and the actual RSSI value, two indicators—location similarity coefficient and close contact distance—can be calculated.
- **Close Contact Judgment:** Based on these two indicators, we design a close contact judgment rule to judge whether two mobile phone users are close contacts. In addition, we consider the time dimension information into the rule and divide close contacts into four levels: very high, high, medium and low.

### 3.2. Location Similarity Coefficient

In general, even if the distance exceeds 100 m, the AP can also receive the probe requests sent by the phone. In addition, thanks to the deployment of multiple APs in the office building, it is possible to obtain probe requests from each AP, which include the time stamp, MAC address and RSSI information of a given smartphone. RSSI information fluctuates in the indoor environment, and it is inappropriate to be used to obtain the physical distance between the smartphone and the AP directly. Nevertheless, the magnitude of RSSI information can reflect the distance relationship among the smartphone and different APs, and this relationship can be utilized to extract the location feature for the smartphone. Based on the location features, we design a indicator that is the location similarity coefficient to transfer the smartphone–AP distance relationship into two smartphones’ distance relationship. Then, we will introduce the calculation method of the location similarity coefficient specifically.

**Figure 4:**
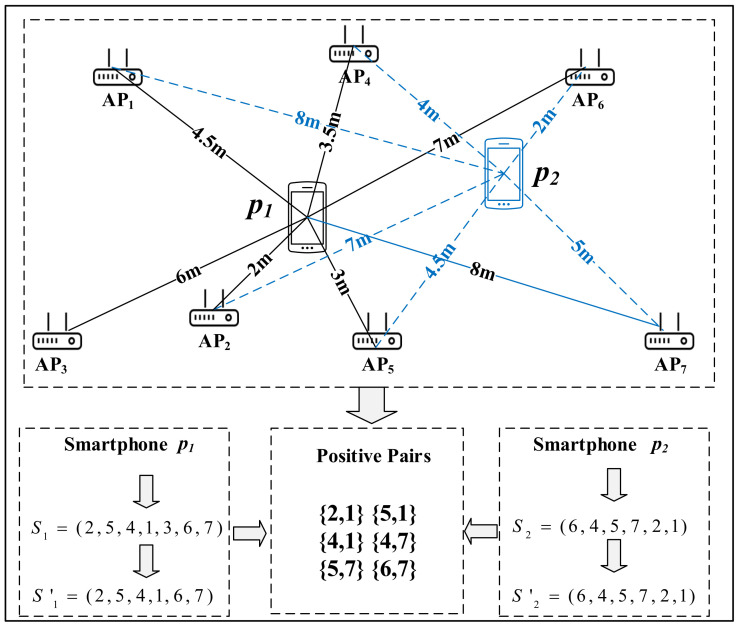
An example.

(1) **Calculate the AP sequence of smartphones**. Without loss of generality, the AP sequence of the smartphone $p_{i}$ is noted as $S_{i}$, which is defined with respect to the RSSI values. After collecting the probe requests from multiple APs during one same time period, we assort them according to the smartphone’s MAC address. For the probe group of smartphone $p_{i}$, we sort the RSSI values in descending order and obtain an AP sequence which is called $S_{i}$. Taking Figure 4 as an example, smartphones $p_{1}$ and $p_{2}$ are surrounded by 7 APs numbered 1 to 7. The AP can detect WiFi probe requests sent by smartphone when it is in the connection range, while some APs are out of the connection area and fail to detect WiFi probe requests. For instance, the $AP_{3}$ is too far to receive the WiFi probe request sent by smartphone $p_{2}$. Thus, the AP sequence of the smartphone $p_{1}$ and $p_{2}$ are $S_{1}=\left(2,5,4,1,3,6,7\right)$ and $S_{2}=\left(6,4,5,7,2,1\right)$, respectively, which is defined with respect to the RSSI values.
(2) **Calculate the number of positive pairs**. First, the intersection set $F_{i,j}$ is defined as the common elements in $S_{i}$ and $S_{j}$. Taking Figure 4 as an example, the intersection set $F_{1,2}$ is defined as the common elements in $S_{1}$ and $S_{2}$. Next, we update the two AP sequences into $S_{i}^{\prime }$ and $S_{j}^{\prime }$, respectively, by retaining elements in $F_{i,j}$. Then, when an AP pair $\left\{ap_{m},ap_{n}\right\}$ appears in $S_{i}^{\prime }$ and it also appears in $S_{j}^{\prime }$ in sequence, the pair of APs $\left\{ap_{m},ap_{n}\right\}$ is regarded as a positive pair. Lastly, $count_{i,j}$ is denoted as the counting number of positive pairs in $S_{i}^{\prime }$ and $S_{j}^{\prime }$.

**Figure 5:**
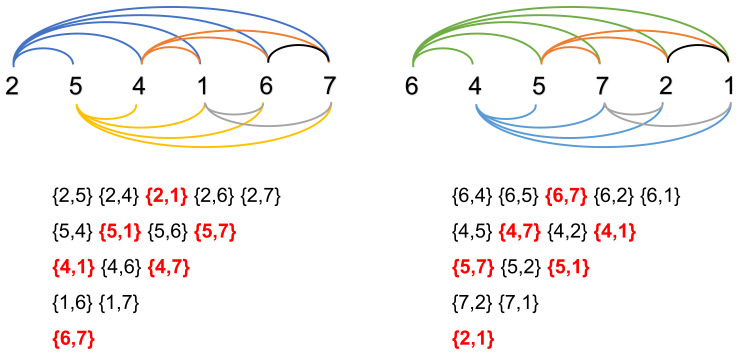
An example of positive pairs.

As shown in Figure 5, we calculate the $F_{1,2}$ of smartphones $p_{1}$ and $p_{2}$ as follows.

(1) $$F_{1,2}=S_{1}\cap S_{2}=\left(1,2,4,5,6,7\right)$$

The $S_{1}^{\prime }$ and $S_{2}^{\prime }$ can be calculated based on the result of $F_{1,2}$.

(2) $$S_{1}^{\prime }=\left(2,5,4,1,6,7\right)$$

(3) $$S_{2}^{\prime }=\left(6,4,5,7,2,1\right)$$

As Figure 4 shows, the positive pairs in $S_{1}^{\prime }$ and $S_{2}^{\prime }$ are {2,1},{5,1},{4,1},{4,7}, {5,7}, {6,7}, that is, $count_{1,2}=6$.

(3) **Calculate the location similarity coefficient**. The location similarity coefficient $lsc_{i,j}$ of smartphones $p_{i}$ and $p_{j}$ can be calculated based on the positive pairs $count_{i,j}$ as follows.

(4) $${\mathrm{lsc}}_{i,j}=\frac{2}{k(k-1)}\times {\mathrm{count}}_{i,j},k>1$$

where *k* is the element number in $F_{i,j}$. The $lsc_{i,j}$ is set to 0.5 at that time k=1. At the moment of k=0, it means that no one AP detects the smartphones $p_{i}$ and $p_{j}$. In this case, we are unable to calculate the $lsc_{i,j}$, resulting in uncertainty as to whether they are close contacts. In the example of Figure 4, for the smartphone $p_{1}$ and $p_{2}$, k=7 and $lsc_{1,2}=2/7\left(7-1\right)\times 6=2/7$.

In case the that the number of APs is small, it is hard to tell whether two smartphones are very close to each other only by the location similarity coefficient. Thus, we introduce another judgment indicator—close contact distance—to determine whether they are close contacts accurately. In indoor environments, especially in the environment of partition walls, RSSI is seriously affected by the multipath effect, but it is relatively accurate in calculating the distance between APs and smartphones based on RSSI. As $AP_{m}$ is the closest AP to the $p_{i}$, and it can be accessed from the AP ordering mentioned in the previous section. Then, we can obtain the distances from the $AP_{m}$ to the smartphone $p_{i}$ and $p_{j}$ based on the free space path loss model of RSSI [18], and they are marked as $D_{i}^{m}$ and $D_{j}^{m}$, respectively. Next, we can calculate the $L_{i,j}^{i}$ as follows.

(5) $$L_{i,j}^{i}=\left|D_{i}^{m}-D_{j}^{m}\right|$$

Furthermore, $L_{i,j}^{j}$ can be calculated in the same way as above. The close contact distance $d_{i,j}$ of smartphone $p_{i}$ and smartphone $p_{j}$ is defined as follows.

(6) $$d_{i,j}=\frac{1}{2}\times \left(L_{i,j}^{i}+L_{i,j}^{j}\right)$$

### 3.3. Close Contact Distance

As shown in the example of Figure 4, we explain the calculation process of the close contact distance between smartphone $p_{1}$ and smartphone $p_{2}$ to elaborate our method clearly. $ap_{2}$ is closest to $p_{1}$ and $ap_{6}$ is closest to $p_{2}$. Thus, $L_{1,2}^{1}=\left|D_{1}^{2}-D_{2}^{2}\right|=5$ m and $L_{1,2}^{2}=\left|D_{1}^{6}-D_{2}^{6}\right|=5$ m. Finally, the close contact distance between $p_{1}$ and $p_{2}$ is $d_{1,2}=\frac{1}{2}\times \left(L_{1,2}^{1}+L_{1,2}^{2}\right)=5$ m.

### 3.4. Close Contact Level

After analyzing with large amounts of experimental statics, we formulate a judgment rule based on the two indicators for determining whether the users of smartphones $p_{i}$ and $p_{j}$ are close contacts. The judgment rule is composed of three conditions:

(1) $d_{i,j}<6$
(2) $lsc_{i,j}>0.5$
(3) $\left\{{\mathrm{lsc}}_{i,j}>0.45\right\}\&\&\left\{d_{i,j}<10\right\}$.

If one of the following conditions is met, the two users can be considered as close contacts. In addition, the time domain information is combined to define a set of fine-grained close contact level, {very high, high, middle, low}. When two people have been in close contact for more than three minutes in the same room, the close contact level is “very high”. When two people have been in close contact for less than three minutes in the same room, the close contact level is “high”. When two people have been to the same building but did not meet up close, the close contact level is “middle”. If they have only been to the same building, the close contact level is “low”. The fine-grained level of close contact can assist governments in making more accurate decisions.

## 4. Evaluation

### 4.1. Settings

#### 4.1.1. Experimental Setup

**Figure 6:**
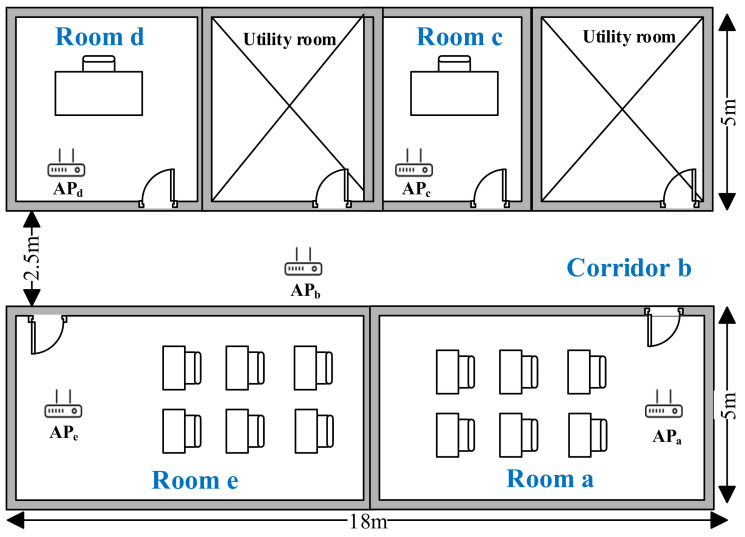
Experimental scenario.

We implemented a great deal of experiments in a college office building. The experimental scene is shown in Figure 6, including two utility rooms and four offices. The total length of all rooms is 18 m and the width is 12.5 m. WiFi probes are collected by 5 APs (Ralink RT5370), APa to APe. In addition, six volunteers with significant differences participated in our experiment to prove the effectiveness of the system. Each volunteer participated in the experiment for more than two hours.

#### 4.1.2. Evaluation Indexes

There are two evaluation indexes as follows.

- True Positive Rate (TP): the proportion of cases in which two close contacts are identified accurately.
- False Positive Rate (FP): the proportion of cases in which two non-close contacts are identified accurately.

### 4.2. Overall Performance

**Figure 7:**
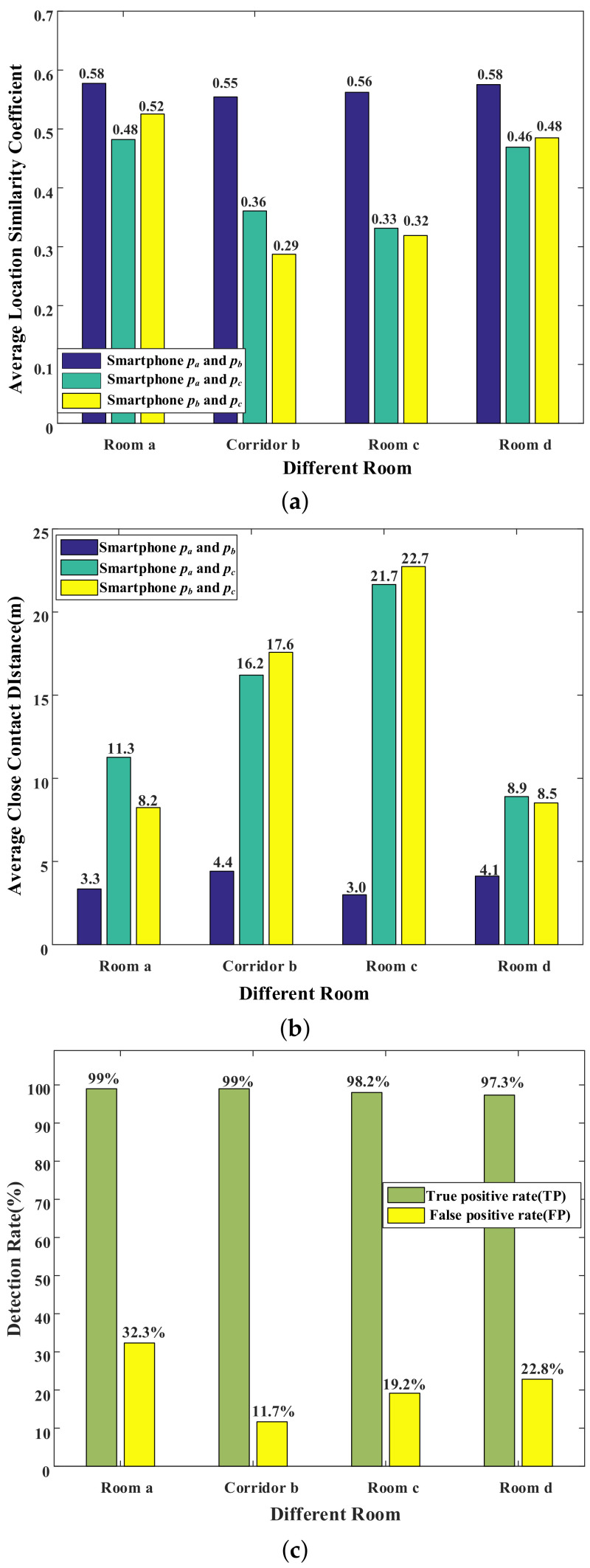
Overall performance including (**a**) location similarity coefficient, (**b**) close contact distance and (**c**) detection rate.

This section will evaluate the overall performance of COVID-19 Tracer. For each experiment, as shown in Figure 6, we invited three volunteers and asked two of them carrying smartphones $p_{a}$ and $p_{b}$ to move casually in Room e, while we asked another person carrying the smartphone $p_{c}$ to move in any other room (e.g., Room a, Corridor b, Room c and Room d). In this condition, volunteers with smartphones $p_{a}$ and $p_{b}$ are regarded as close contacts, while the volunteer with smartphone $p_{c}$ is regarded as the non-close contact. Figure 7 shows the experimental results, in which the location similarity coefficients of smartphones $p_{a}$ and $p_{b}$ are all greater than 0.5, and the contact distances of smartphones $p_{a}$ and $p_{b}$ are closer than other smartphone pairs. It can be inferred that our designed indicators perform well in representing two close contacts. In Figure 7c, it can be found that the average TP is more than 98% and the average FP is less than 25%, resulting in a conclusion that our system does have good performance in judging close contacts.

### 4.3. Effect of Various Factors

#### 4.3.1. Effect of Distance

**Figure 8:**
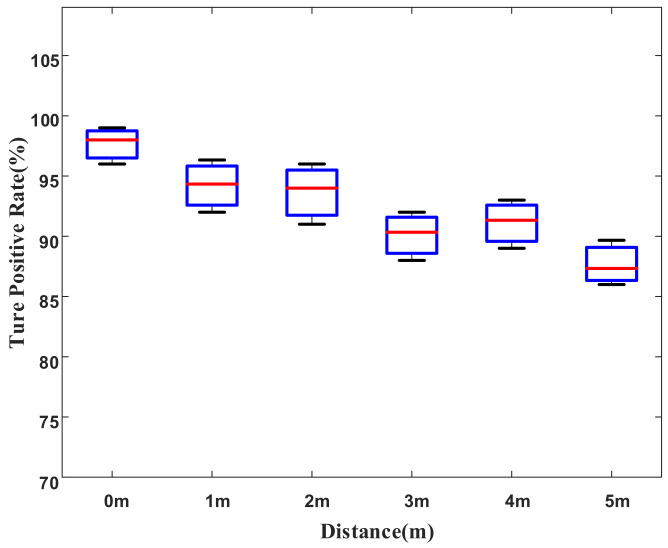
Effect of distance.

We evaluate the impact of the distance between smartphones on the system performance. In the scenario of the college building with 5 APs, we placed two smartphones in the same room and broadened the distance between two smartphones from 0 m to 5 m in steps of 1 m. We conducted this group of experiments every day for two weeks and calculated the average TP values under each distance. As shown in Figure 8, the TP was about 98% when two smartphones were next to each other, that is, the distance was 0 m. Besides, the system performance shows a decreasing trend as the distance increases. However, the TP can reach more than 85% even at a distance of 5 m.

#### 4.3.2. Effect of AP Number

**Figure 9:**
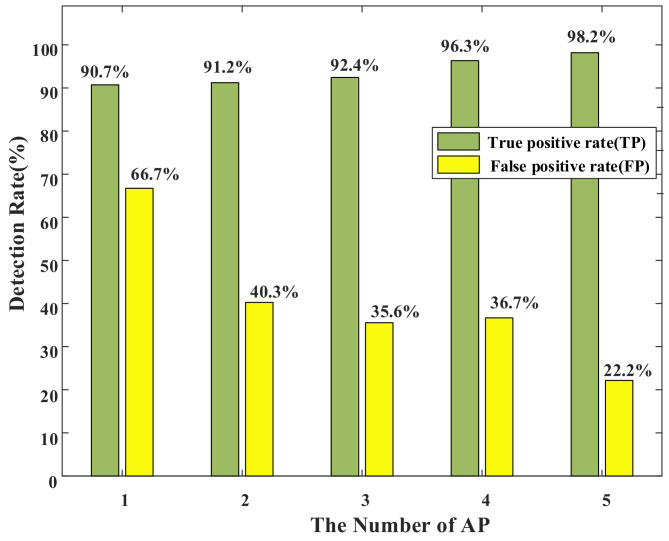
Effect of AP number.

To evaluate the impact of the number of APs on system performance, we deployed several APs ranging from 1 to 5 in steps of one in the same experimental scenario. A large number of data were collected within two weeks, and the results are shown in Figure 9. In this figure, it can be seen that TP increased with the increase in AP number, and the TP can reach more than 90% in all cases. The FP of the system shows a clear downward trend as the number of APs increases. In summary, we can conclude that the more APs there are, the better the performance of the system. It is worth noting that even with only one AP, our system can perform very well.

#### 4.3.3. Effect of Personnel Density

**Figure 10:**
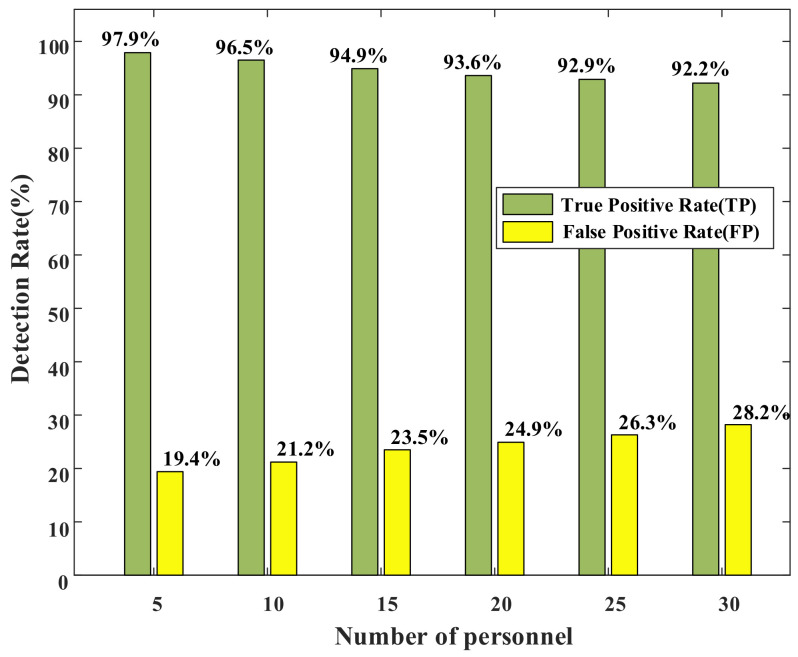
Effect of personnel density.

We consider that when there are many people in an environment, the human body’s occlusion of the signal will also affect our system. To evaluate the impact of personnel density on the system, in Room e, we tested the system performance with the number of people at 5, 10, 15, 20, 25 and 30. As shown in Figure 10, as the number of people in the scene increases, the system’s TP gradually decreases. Nevertheless, even if the number of people reaches 30, the TP of the system still reaches 92.7%. Furthermore, the FP of the system gradually increases as the number of people increases. However, all remained below 30%.

Therefore, we can claim that our system can still function normally in an environment with high personnel density.

### 4.4. Evaluation of Special Environments

This section will evaluate the performance of the COVID-19 Tracer in some special environments. Suppose there are two people in an adjacent room with a separated wall (i.e., the locations of A and B in Figure 2). In this case, they are not close contacts, but the short distance may induce the system to make a wrong judgment. In this situation, we collected a lot of data and found that the average FP is 15.6%, which is an acceptable result. In addition, we also studied the effectiveness of the system in another scenario, where five APs are deployed on the upper and lower floors of a building, and two smartphones are placed in different positions in the same room on the upper and lower floors. In this case, the average value of FP is 13.3%. The experimental results show that the COVID-19 Tracer can still function normally even under some special environments.

## 5. Prototype System

**Figure 11:**
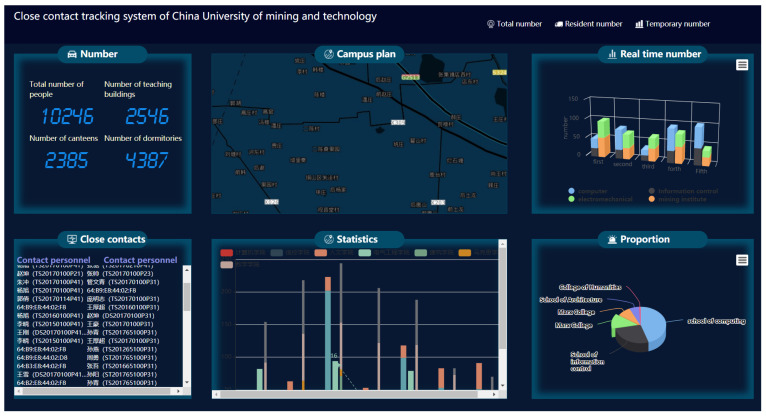
Prototype system.

With our proposed COVID-19 Tracer system framework, we constructed a prototype system. This system’s main interface is shown in Figure 11, which can be deployed in an office building or a university. It includes the following four modules.

- **Personnel information management module**

This module is mainly used to manage the information (such as name, MAC address and mobile phone number) of regular personnel, where regular personnel refers to company employees or university students.

- **Close contact inquiry module**

This module is mainly used to query the close contacts of the patient. In this module, we enter the patient’s mobile phone MAC and then click the query button. The system will display the information of all the people who have been in contact with him or her in the past 14 days.

- **Patient track query module**

This module is mainly used to query the movement trajectory of a patient and what room they have been to.

- **Information statistics module**

This module is mainly used to count the number of people in a room or a building.

**Figure 12:**
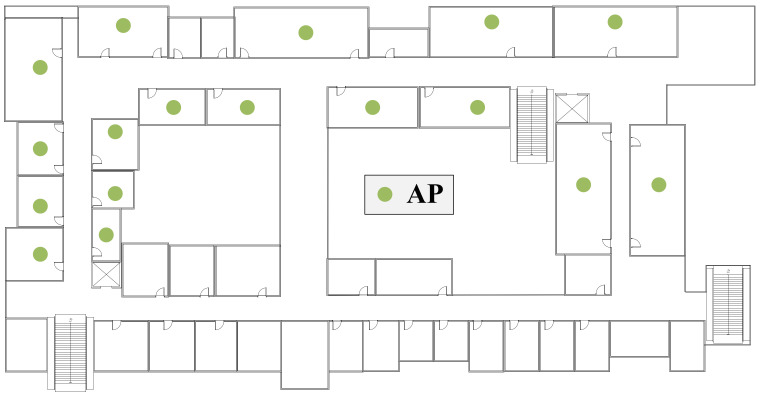
Prototype system deployment scenario.

To test the performance of the prototype system, this system was deployed on the fifth floor of the computer school of China University of Mining and Technology. Figure 12 shows the prototype system deployment scenario.

**Figure 13:**
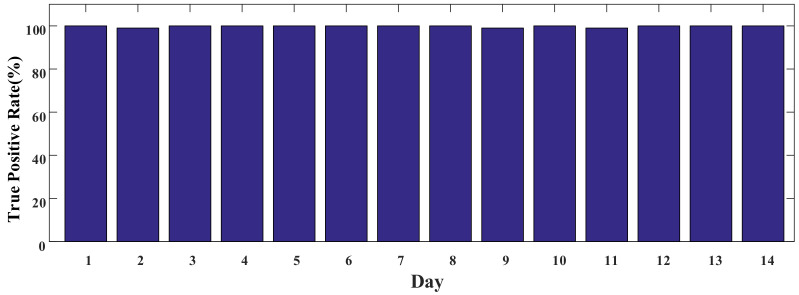
Prototype system performance.

We deployed an AP in each graduate student’s office room to collect the users’ MAC addresses. Then, 50 graduate students working on this floor were invited to participate in our experiment. The MAC addresses of their smartphones were entered into the system. Moreover, we asked them to turn on WiFi every day. In the system, we selected 100 pairs of close contacts who worked in the same room every day. We also selected each pair of close contacts and checked whether the two people were close contacts through their MAC addresses in our system. The TP of our system was counted over 14 days. Figure 13 shows that the TP exceeded 99%.

## 6. Discussion

In this section, we discuss some potential issues in the practical application of the system and the corresponding solutions.

**How to send notifications to close contacts?**

After identifying a close contact by COVID-19 Tracer, we can only record the MAC address of the close contact’s smartphone, while sending notifications to the close contact is still a future work. The first feasible method is to integrate the search results into a health code. This code is an epidemic prevention measure involving all people in China as the government demands everyone to create the health code on their smartphone. A health code in green color means you are safe, while a health code of red means you may be exposed to danger. Once a person has close contact with a COVID-19 patient, his or her health code will turn to red. The second feasible method is to build an information-referring system based on the search results. In the event of an epidemic, people can inquire and verify if they are close contacts by entering the MAC addresses of their smartphones in the query system.

- **How to make sure that all close contacts are not missed?**

In actual situations, it is hard to obtain the information when some people do not turn on WiFi, so we consider using computer vision technology to improve the system. The camera and APs are installed at the entrance of the building, so if we do not detect a WiFi probe, we will take pictures and record photos of people. Once the epidemic occurs, these photographed people can be treated as potential close contacts and we may provide the government with information on potential close contacts to assist them in making better decisions.

- **How to deal with random MAC addresses?**

To protect user privacy, some smartphones use MAC address randomization technology, which is a random, anonymous device identifier instead of a real address when the device connects to a wireless network. Therefore, the random MAC addresses are sniffed by the wireless router when some smartphones send WiFi probe request packets.

Fortunately, we found that the random MAC addresses will not change after the connection is established. Besides, when connecting to the same wireless network access point multiple times, the same random MAC address is used. According to this feature, our system can still trace this user based on this random MAC address. To address this MAC randomization problem, we can use wireless roaming technology to cover the same wireless access point in a university or office building. Therefore, once a user has connected to this access point, this user will automatically connect to this wireless access point across the entire university or office building, and his MAC address will not change. Afterward, our system can utilize this MAC address to track this user.

- **How to solve privacy issues?**

The legality of WiFi detection technology is also controversial as some works reveal that WiFi technology can be used to detect a person’s vital signs and activity information in a passive way. However, from the investigations results on different groups of people, it is inferred that people generally do not think that data grabbed from open wireless networks will leak personal privacy. Moreover, it is considered acceptable to utilize WiFi probes to assist the government in its epidemic prevention work.

## 7. Related Work

### 7.1. WiFi Probe Technology

WiFi probe technology utilizes the RSSIs, timestamps, MAC addresses and service set identifiers (SSIDs) contained in probe requests to design various application systems, such as crowd counting [21,22], passive tracking [23,24,25] and analysis of equipment utilization [26,27]. Different from other solutions, the probe-request-based solution has the characteristics of low-cost and passive data collection. Other works in the literature [28,29,30,31] have used the similarity of SSIDs to detect the intimacy of different smartphones.

Nevertheless, 80% of the devices reported that the SSID list is empty [32,33], which causes the SSID-based scheme to not work. Therefore, most of the works based on WiFi probe technology use RSSI information. Refs. [34,35] use spatial features and pseudo-spatial features extracted from signal strength to detect pedestrians. However, because RSSI-based solution accuracy is not high enough, the performance of spatial features is not reliable. Refs. [36,37] consider the diversity of hardware, and the normalized RSSI vector is used to detect occupancy patterns, proximity relationships and social interactions. However, the transmission time of the probe is determined by the internal mechanism of the hardware, and different devices may generate different data granularity, which makes the similarity comparison work challenging.

### 7.2. WiFi-Based Passive Tracking

In recent years, WiFi-based passive tracking has become a research hotspot, which uses the target movement’s reflected signal characteristics to track the target without carrying any equipment. The WiFi-based solution is a promising solution because WiFi signals are everywhere. The WiFi-based solution uses the reflection of the target’s movement on the WiFi signal to extract the target’s position and movement information. WiFi signal parameters include angle-of-arrival (AoA) [38,39,40], time-of-flight (ToF) [41,42,43] and Doppler frequency shift (DFS) [43,44,45]. WiDar [46] uses the DFS of the signal to calculate the target’s position and velocity.

However, WiDar cannot calculate the position independently due to the lack of phase information, and there is a cumulative error. IndoTrack [44] combines AoA with DFS, which can be used for continuous tracking. Widar2.0 [47] uses a maximum likelihood algorithm for joint signal parameter estimation, which can be tracked based on a single link. mD-Track [45] proposes a path separation method that can extract the reflected signal of the target of interest. WiFi-based passive tracking is not suitable for searching for close contacts due to these solutions working poorly in multi-person tracking scenarios. Additionally, in close contact tracing, we need to track a person’s location and know who he or she is. Therefore, we need to utilize other technologies to search for close contacts.

## 8. Conclusions

This paper creatively proposes a low-cost passive detection system that utilizes WiFi probe technology to identify close contacts of COVID-19 cases. We present two novel judgment indexes for close contacts—location similarity coefficient and close contact distance—to design a range-free judgment algorithm for position similarity. Then, we propose a novel fine-grained classification scheme for the risk level of close contacts according to two indexes. Extensive experiments were conducted in a school office building, and the average recognition accuracy of close contacts was over 98%. Finally, we have developed a prototype system that provides a visual interface for the display of search results. In addition, we systematically compared our study with other works and summarized the focus and direction of our next work.
